# Enhanced LoRaWAN Adaptive Data Rate for Mobile Internet of Things Devices

**DOI:** 10.3390/s20226466

**Published:** 2020-11-12

**Authors:** Arshad Farhad, Dae-Ho Kim, Santosh Subedi, Jae-Young Pyun

**Affiliations:** Wireless and Mobile Communication System Laboratory, Department of Information and Communication Engineering, Chosun University, Gwangju 61452, Korea; arshad@chosun.kr (A.F.); wireless@chosun.kr (D.-H.K.); santoshmsubedi@chosun.kr (S.S.)

**Keywords:** LoRaWAN, adaptive data rate, mobility, Internet of Things, resource assignment, convergence period

## Abstract

A long-range wide area network (LoRaWAN) is one of the leading communication technologies for Internet of Things (IoT) applications. In order to fulfill the IoT-enabled application requirements, LoRaWAN employs an adaptive data rate (ADR) mechanism at both the end device (ED) and the network server (NS). NS-managed ADR aims to offer a reliable and battery-efficient resource to EDs by managing the spreading factor (SF) and transmit power (TP). However, such management is severely affected by the lack of agility in adapting to the variable channel conditions. Thus, several hours or even days may be required to converge at a level of stable and energy-efficient communication. Therefore, we propose two NS-managed ADRs, a Gaussian filter-based ADR (G-ADR) and an exponential moving average-based ADR (EMA-ADR). Both of the proposed schemes operate as a low-pass filter to resist rapid changes in the signal-to-noise ratio of received packets at the NS. The proposed methods aim to allocate the best SF and TP to both static and mobile EDs by seeking to reduce the convergence period in the confirmed mode of LoRaWAN. Based on the simulation results, we show that the G-ADR and EMA-ADR schemes reduce the convergence period in a static scenario by 16% and 68%, and in a mobility scenario by 17% and 81%, respectively, as compared to typical ADR. Moreover, we show that the proposed schemes are successful in reducing the energy consumption and enhancing the packet success ratio.

## 1. Introduction

Low-power wide-area networks (LPWANs) are a growing technology that target static and mobile Internet of Things (IoT) applications requiring long-range, energy-efficient, and low data rate communication [1]. A long-range wide area network (LoRaWAN) [2] is an LPWAN technology that has been widely adopted as a substitute for IoT applications [3]. IoT applications can be categorized into nine groups: smart metering, agriculture (e.g., crop monitoring), tracking (e.g., logistics and pet location), smart grids, health, industrial, smart city, home automation, and vehicle telematics [4]. These applications have strict requirements, including the packet length, packet success ratio (PSR), reliability, and mobility characteristics [5]. LoRaWAN provides effective solutions in order to meet a wide range of IoT application requirements.

**Figure 1:**
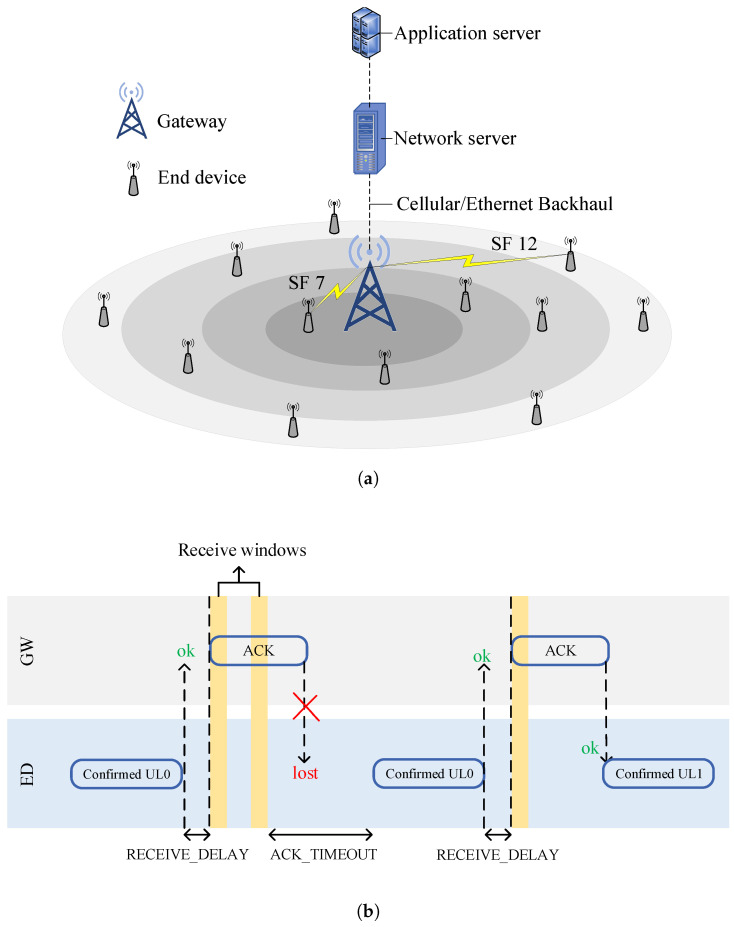
A brief working of long-range wide area network (LoRaWAN): (**a**) underlying architecture comprised of end devices (EDs), gateway (GW), network server (NS), and application server, and (**b**) transmission procedure in confirmed mode.

The LoRaWAN end devices (EDs) are classified as class *A*, class *B*, and class *C* [6]. Among them, class *A* EDs deal with sensors and are implemented in IoT applications, owing to their energy efficiency and bi-directional communications. Figure 1a shows a LoRaWAN network, where EDs describing IoT nodes always transmit packets to a gateway (GW) while using spreading factors (SFs) ranging from seven (SF7) to twelve (SF12) in the uplink (UL). The GW is connected through other communication technologies (e.g., Cellular (3G/4G), Ethernet) to a centralized network server (NS) responsible for functions such as traffic management [7]. The NS is deployed in a cloud computing environment (rarely co-located near the GW [8]), accountable for collecting data from the GW and sending them to appropriate applications. Therefore, in the case of delay-sensitive applications, edge and fog computing solutions have also been studied in order to deploy LoRaWAN network components [9,10], bringing services, such as advanced analytics and distributed storage, closer to the EDs [11]. In class *A*, every UL transmission is followed by two receive windows, namely RX1 and RX2, for receiving a downlink (DL) acknowledgment (ACK) from the NS, as highlighted in Figure 1b. RX1 uses the same SF and channel set as the UL transmission, whereas the RX2 uses a dedicated channel (i.e., 869.525 MHz) and a fixed SF (i.e., SF12) [12]. If an ACK is not received in both, receive windows, a random delay, i.e., ACK_TIMEOUT, is added before a retransmission is conducted in order to avoid consecutive collisions. To this end, an ED chooses a random time between 1 and 3 s and applies a retransmission [13].

Moreover, the class *A* EDs of LoRaWAN employ an adaptive data rate (ADR) mechanism at both the ED and NS [14]. The ED-managed ADR is only responsible for incrementing the SF. At the same time, NS-managed ADR adjusts both the SF and transmit power (TP) after receiving *M* (*M* = 20 is used [15] for the experiments and discussion in this study) UL packets from the ED based on the prevailing channel conditions. However, the performance of the ADR in terms of the PSR is severely affected by the variable channel conditions [16]. Therefore, the typical ADR suffers from a high convergence period, owing to the time-consuming process of both ED- and NS-managed ADRs. The typical ADR scheme requires several hours to days to converge into a stable and energy-efficient communication state when the NS starts monitoring the *M* packets. During the convergence period, the typical ADR suffers from a massive packet loss caused by interference and packets arriving under the pre-defined sensitivity at the GW. The convergence period for static EDs was studied under variable channel conditions in [17], where it was revealed that ADR suffers from a high convergence period when the link quality degrades, and EDs want to move from a lower to higher SF or TP to recover their connectivity. Furthermore, the authors in [18] proposed some changes in a typical ADR, where it was suggested to change the SF and TP of the individual ED after five UL packets (in a typical ADR, this UL history is set to *M* packets) under a static ED environment. Therefore, in order to reduce the convergence period and improve the PSR (for both static and mobile IoT EDs) in a confirmed mode, we propose two NS-managed ADRs and claim the following contributions.

1. First, we propose a Gaussian filter-based ADR (G-ADR) to smooth the signal-to-noise ratio (SNR) of *M* packets received at the NS. Through real-time experiments and computer simulations, we show that the SNR of LoRaWAN packets received at an NS follows a Gaussian distribution. By employing a Gaussian filter, G-ADR can optimally find both SF and TP parameters, which results in a reduced convergence period and improved PSR.
2. Second, we propose another NS-managed ADR based on the exponential moving average (EMA-ADR). Through computer simulations, we show that the smoothing process using the EMA filter decreases the spikes of raw SNR values. Hence, EMA-ADR advances the PSR and reduces the convergence period when compared to the typical ADR.
3. In addition, we show that both G-ADR and EMA-ADR, when jointly utilized with the initial SF allocation method, significantly improve the convergence period and PSR.

The rest of the paper is organized, as follows: Section 2 provides an overview of the published literature comprised of an enhancement and reduction of the convergence period in a typical ADR. Section 3 elaborates on the proposed schemes. Section 4 presents the experimental results and an analysis of the proposed schemes in comparison with the typical ADR. Section 6 presents some concluding remarks.

## 2. Related Studies

Many studies have been conducted in order to solve the problems of typical ADRs in terms of the convergence period, scalability, and PSR. In particular, the literature is focused on the enhancement [19,20,21,22] and convergence period reduction of a typical ADR [17,18].

### 2.1. Enhancements in Typical ADR

In [19], an approach to avoiding unnecessary changes to the SF that occur at the NS-managed ADR is presented. Their method offers a congestion classifier, which determines whether to switch to a higher SF or adjust the back-off time to avoid massive congestion. The congestion classifier is based on UL and DL packets. If the number of DL packets received by the ED is equal to the number of UL packets, then no congestion is shown. Otherwise, the SF is reduced because a higher SF is vulnerable to interference owing to a high time-on-air. When an ED fails to receive an ACK after ADR_ACK_DELAY, it chooses a long back-off time. By contrast, it increases the SF to extend the network scalability and coverage. The authors showed that their method reduces the delay because the ED maintains the SF and increases the back-off time when congestion occurs.

An ADR+ scheme was proposed in [20] in order to improve the ADR performance. The ADR+ scheme slightly modifies the NS-managed ADR by taking the average SNR of the last *M* packets received. Thus, the ADR+ plays a vital role in increasing the consistency and energy efficiency of EDs under variable channel conditions. ADR+ shows an improved performance in terms of PSR and energy consumption when compared to a typical ADR.

It should be noted that a typical ADR is inadequate and inefficient when the EDs are mobile, because it manages the SF and TP after the reception of *M* packets [21]. In addition, the typical ADR does not consider the degradation of the received signal that is caused by the ED mobility, the presence of building penetration loss, or moving objects. Therefore, Ref. [21] suggests a network-managed enhanced ADR (E-ADR). The E-ADR is primarily based on the trilateration technique to estimate a mobile ED’s next position with a pre-defined trajectory. An experiment using E-ADR as compared to a typical ADR was conducted by applying five EDs and three GWs in a testbed scenario. The authors claim that NS quickly defines SF and TP, which results in low energy consumption and packet loss.

In [22], the authors suggest another enhanced ADR with coding rate adaptation in the LoRaWAN under the unconfirmed mode of LoRaWAN. The primary aim is to improve the tradeoff between the PSR and energy consumption, which considers the coding rate and the capture effect, by taking the average of *M* packets to fine-tune the link performance of the EDs. The method that is described in [22] outperforms a typical ADR in terms of energy consumption and fairness.

By summary, the methods that are presented in [19,20,21,22] enhanced the performance of a typical ADR. The congestion classifier-based method described in [19] is only focused on improving the data rate adaptation. However, it ignores the TP adaptation because the energy consumption in LoRaWAN is mainly based on the TP and the SF [23]. In [20], ADR+ was evaluated only in a small environment for static EDs. E-ADR described in [21] was considered for mobile EDs; however, it is solely based on a pre-defined trajectory of the ED. Finally, the method in [22] was compared to a typical ADR in unconfirmed mode. Note that a typical ADR performance is significantly affected by bi-directional communication supporting both the UL and DL. Keeping these facts in mind, we propose two NS-managed ADRs to enhance ADR performance in terms of the convergence period, PSR, and energy consumption.

### 2.2. Reduction of Convergence Period in Typical ADR

A performance assessment of the convergence period under different configurable parameters was conducted in [17]. This study provides in-depth insight into the typical ADR under variable channel conditions by highlighting limitations, such as the convergence period. The simulation results showed that the ADR convergence period and energy consumption are primarily dependent on the link conditions and number of EDs. It was revealed that the ADR suffers from a high convergence period under variable channel conditions and when the EDs change their SF or TP to a higher value (such as SF = 12 and TP = 14 dBm) to recover their connectivity with the GW. Furthermore, the convergence period is more sensitive to ADR_ACK_DELAY than ADR_ACK_LIMIT (i.e., ADR_ACK_DELAY and ADR_ACK_LIMIT are equal to 32 and 64 UL packets in the ED-managed ADR, respectively). Their simulation results also revealed that the typical ADR convergence period introduces higher energy consumption and more significant packet losses.

It was recently indicated that typical ADRs (both ED- and NS-managed) suffer from convergence issues [18]. An ED-managed ADR is inefficient for lossy links, which results in considerable time to converge to a constant and stable state. Meanwhile, the NS-managed ADR takes *M* packets to alter the SF and TP, which makes it too time-consuming to determine a reliable configuration. Therefore, Ref. [18] suggests some changes (such as decreasing *M* packets during SF and TP configuration adaptation) in both ED- and NS-managed ADRs for enhancement. NS-managed ADR controls the ED-managed ADR by computing the PSR of an individual ED before sending the DL packet (LinkADRReq) in response to ADRACKReq. Here, the PSR is compared to a predefined threshold (i.e., PSR < 80%). If the condition holds, NS sends the LinkADRReq MAC layer command for the ED containing the SF and TP. Indeed, it changes the SF and TP of the individual ED after five UL packets (in a typical ADR, this UL history is set to *M* packets). The performance of both enhanced methods (ED- and NS-managed ADRs) has been compared to typical ADRs, where the results show improved outcomes in terms of the convergence period, energy consumption, and PSR. However, the performance evaluation of the enhanced methods is limited to static EDs and it only a confirmed mode of communication. By contrast, we consider mobility under intra-SF interference and different propagation loss models (such as log-distance and shadowing losses).

## 3. Proposed ADR Schemes

**Figure 2:**
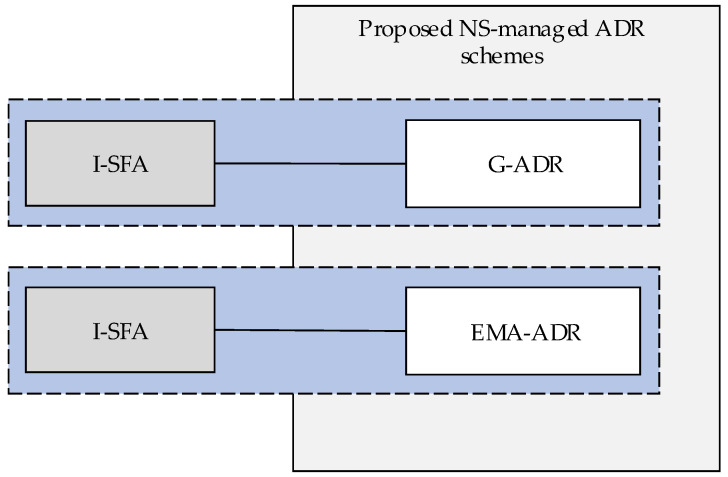
Overview of the proposed NS-managed ADR schemes.

Figure 2 shows an overview of the proposed NS-managed ADR schemes. Note that we do not modify the ED-managed ADR method, but the NS-managed ADR operation is replaced with the proposed methods.

**Figure 3:**
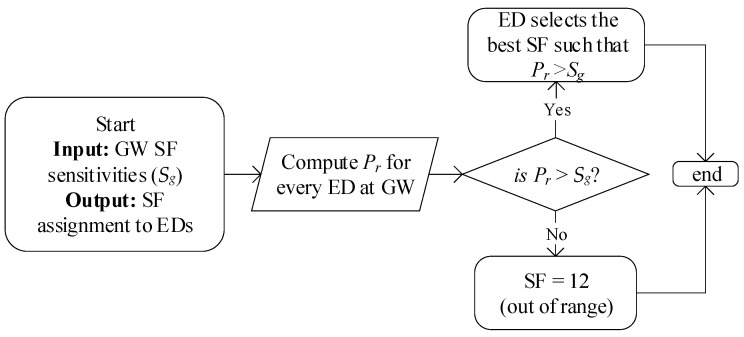
Working procedure of I-SFA scheme [24].

As indicated in Figure 2, we use an initial SF allocation (I-SFA) scheme [24] during the deployment phase jointly with the proposed methods. The primary aim of the I-SFA scheme is to allocate the SF to the EDs during the initial deployment based on the received signal strength ($P_{r}$) at the GW (i.e., a GW would receive from the ED). The I-SFA scheme does not follow a fixed-width SF assignment operation, owing to the variable channel conditions that are caused by shadowing and fading. The working procedure of the I-SFA scheme is shown in Figure 3, where each ED computes $P_{r}$ at the GW. Based on $P_{r}$, the SFs are allocated, such that $P_{r}$ is always higher than each SF sensitivity ($S_{g}$, as shown in Table 1), thereby lowering the interference and avoiding packet loss arriving from the EDs under the sensitivity at the GW.

### 3.1. Gaussian Filter-Based ADR (G-ADR)

**Figure 4:**
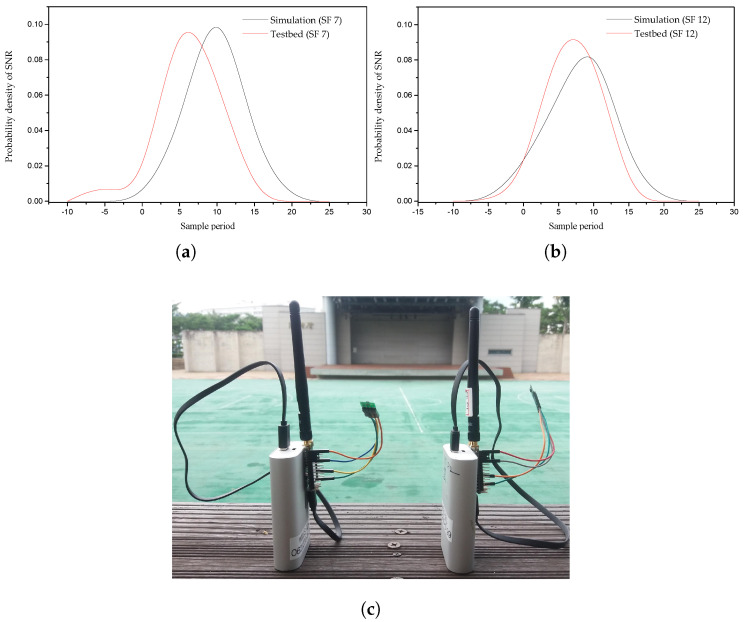
The probability density function (PDF) of the SNR of *M* packets received at the network server using real-time experiment and computer simulation: (**a**) PDF of SNR using SF7; (**b**) PDF of SNR using SF12, and (**c**) deployment of two EDs with SF7 and SF12.

The signal strength that is received at the GW can be thought of as a Gaussian distribution [27]. For example, we show through real-time experiments and computer simulations that the SNR received at the NS follows a Gaussian distribution using SF 7 and SF 12, as shown in Figure 4a,b, respectively. Therefore, we propose the use of a Gaussian filter in order to estimate the value of the SNR to accurately compute SF, TP, or both because these parameters (SF and TP) are dependent on the SNR. Through a computer simulation, we computed the received signal strength based on a link measurement model, which considers various aspects, such as TP, shadowing, fast fading, and antenna gains, as shown in [6]. The steps that are involved in the proposed G-ADR scheme are as follows:

1. When the NS receives an UL packet with the ACK bit enabled in the frame header of the ADRACKReq MAC command, the NS starts tracking the SNR of the *M* received packets. The G-ADR algorithm is initiated by computing the mean (μ) and variance (σ) using (1) and (2) [28], respectively.
  (1) $$\mu =\frac{1}{M}\sum_{i=0}^{M}SNR_{i},$$
  (2) $$\sigma^{2}=\frac{1}{M-1}\sum_{i=0}^{M}{\left(SNR_{i}-\mu \right)}^{2},$$
  where *i* is the *i*th packet among *M*, where *M* = 20.
  Now, the probability density function (PDF) is expressed, as follows [28]:
  (3) $$\;f\left(SNR\right)=\frac{1}{\sigma \sqrt{2\pi }}e\frac{-{\left(SNR-\mu \right)}^{2}}{\sigma^{2}}.$$
2. The proposed G-ADR accepts the centralized SNR values that lie within the effective range of μ + σ and μ − σ. The SNR value is estimated by averaging the values that are within the effective range.
3. Finally, the G-ADR obtains the SNR required ($SNR_{req}$, a demodulation threshold based on the current data rate (DR), as shown in Table 1) and computes the SNR margin ($SNR_{margin}$) and $N_{step}$ while using (4) and (5) [17], respectively.

(4) $$SNR_{margin}=SNR_{m}-SNR_{req}\left(DR\right)-margin_{dBm},$$

(5) $$N_{step}=int\left(\frac{SNR_{margin}}{3}\right).$$

In (5), $N_{step}$ represents the number of times the algorithm is executed [17]. When $N_{step}$ is 0, the ED is already using the best possible configuration condition. If $N_{step}$ is greater than 0, it indicates that there is still a reasonable margin to optimize the configurable parameters. First, the number of SFs is decreased until it reaches a minimum limit (note that NS-managed ADR does not increase the SF, and only the ED-managed ADR is responsible for increasing this number). Second, the TP is decreased by 2 until it reaches a minimum limit (i.e., 2 dBm). Finally, when $N_{step}$ is negative, only the TP is increased by 2 until the maximum limit is reached (i.e., 14 dBm). These parameter (SF and TP) settings are transmitted to the corresponding ED while using the LinkADRReq MAC command as unconfirmed (i.e., NS requires no ACK notification from the EDs). Algorithm 1 describes the detailed operation of the G-ADR method.

### 3.2. Exponential Moving Average-Based ADR (EMA-ADR)

An EMA is a type of weighted moving average, which refers to a weighting factor for each SNR value of *M* packets. In general, the SNR varies over time, even in a fixed environment, resulting in an inaccurate SF and TP configuration [16]. The reasons why the SNR shows such high variability in space and time include various noise factors, fading, interference, and attenuation. The EMA for time-series data can be computed iteratively, as follows:

(6) $$S_{t}=\left\{\begin{array}{ccc} \; & Y_{t}, & t=1\\ \; & \beta .Y_{t}\;+\;(1\;-\;\beta ).\;S_{t-1}, & t>1 \end{array}\right..$$

In (6), $Y_{t}$ represents the current SNR value at time *t*, $S_{t}$ denotes the value of the EMA at any time *t*, and β is a smoothing factor (0 < β < 1). Note that a larger value of β reduces the level of smoothing, whereas a value of β close to zero has a greater smoothing effect and it is less responsive to recent SNR observations. Therefore, we used β = 0.7, because of the mobility feature (Ref. [29] uses β = 0.5 for indoor positioning).    
**Algorithm 1:** Proposed Gaussian filter-based adaptive data rate (G-ADR) scheme.
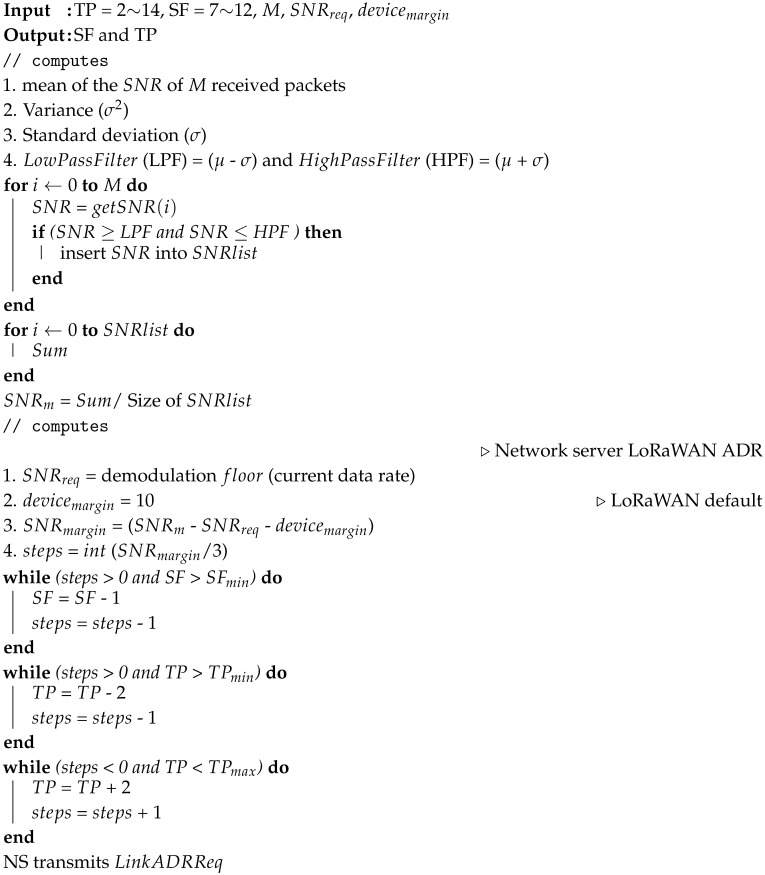


**Figure 5:**
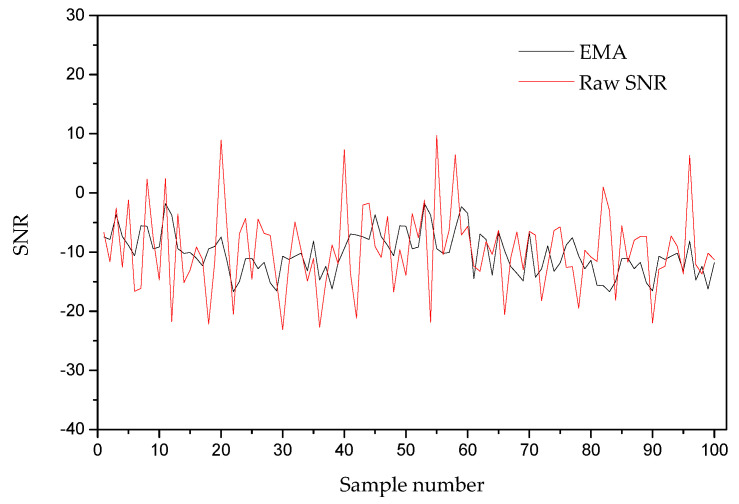
Smoothed SNR using exponential moving average-based (EMA) filter.

In terms of SF and TP estimation while using the measured SNR, an SNR smoothing process is required. Therefore, we conducted an EMA filter-based smoothing of the SNR for several packets received at the NS through a computer simulation, as shown in Figure 5. It can be seen that the smoothing process reduced the spikes of the raw SNR values. In addition, the smoothing operation in EMA smoothing can yield results with only the first two SNR observations [29].

It was recently shown in [20] that, by taking the average of *M* packets, one can improve the results in terms of the PSR and lower the energy consumption when compared to a typical ADR. Therefore, we propose using the EMA filter for SNR smoothing, which resists the rapid changes in the SNR of *M* packets and acts as a local averaging function, and it is directly related to the β parameter. The proposed EMA-ADR can improve the performance in terms of the PSR, convergence period, and energy consumption.

## 4. Experimental Results and Analysis

In this section, we present a comprehensive performance assessment of the proposed schemes, which are examined in comparison with typical ADR and ADR+ [20] in terms of the convergence period, PSR, and energy consumption in both static and mobility scenarios in a confirmed mode. The simulation experiments were performed while using an NS-3 [30].

### 4.1. Simulation Setup

We consider class *A* EDs (*N*), uniformly distributed around a single GW within a 6-km radius. These EDs follow the frequency regulation of the European region, where the UL duty cycle of the EDs and GW is limited to 1% and 10%, for the default channels, respectively. The GW and ED antenna heights are set to 15 and 1.5 m, respectively. EDs mobility follows a random walk two-dimensional (2-D) mobility model. EDs choose a random speed of between 0.5 to 1.5 m/s (in [31], 2 m/s is used for outdoor positioning) and changes direction after every 1000 m [32]. Every ED transmits λ packets/day during four days of the simulation time, where the results are generated using an average of 10 simulations with different seeds. Table 2 presents the rest of the simulation parameters.

### 4.2. LoRaWAN Network Environment

#### 4.2.1. Initial Network Topology

**Figure 6:**
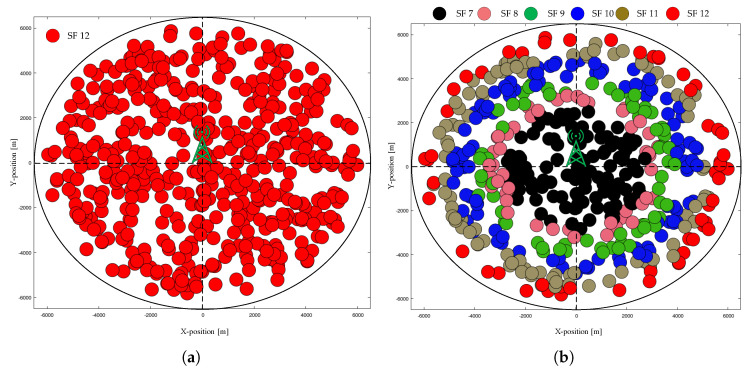
Initial network topology in the case of static EDs with N = 500: (**a**) starting SF = 12, and (**b**) starting SF = I-SFA.

During the initial deployment, SF = 12 is assigned to the EDs as the initial SF in all schemes [18]. In some cases, the initial SF allocation is based on I-SFA (in the case of only the proposed methods). The initial simulation environments with SF = 12 and I-SFA deployment are shown in Figure 6a,b, respectively.

#### 4.2.2. Final Network Topology

**Figure 7:**
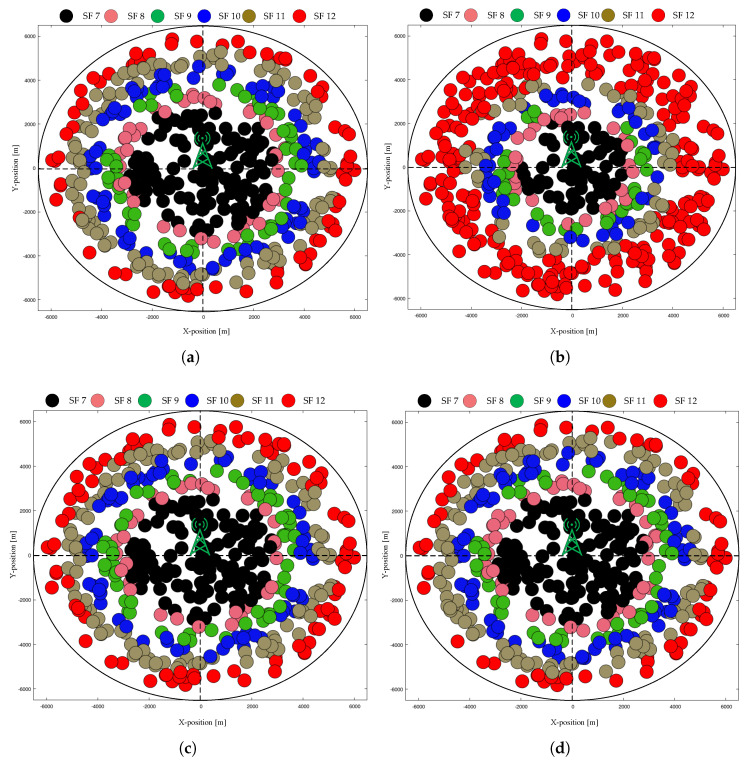
Final network topology in the case of static EDs with N = 500 and starting SF = 12: (**a**) typical ADR; (**b**) ADR+; (**c**) G-ADR, and (**d**) EMA-ADR.

Figure 7 shows a top-down view of the network, with a single GW located at the center and the location of individual EDs marked according to the final stable SF of each ED. In Figure 7, all of the EDs start transmitting data in the UL using SF = 12, where the number of EDs is 500. Table 3 lists the final SF assignment percentages of static EDs at convergence for those that are shown in Figure 7.

### 4.3. Convergence Period Analysis

In this paper, we define the convergence period as the amount of time until the EDs in the network reach a steady SF and PSR. The convergence period is highly dependent on both the initial SF and number of EDs in the network [18].

#### 4.3.1. Static EDs scenario

**Figure 8:**
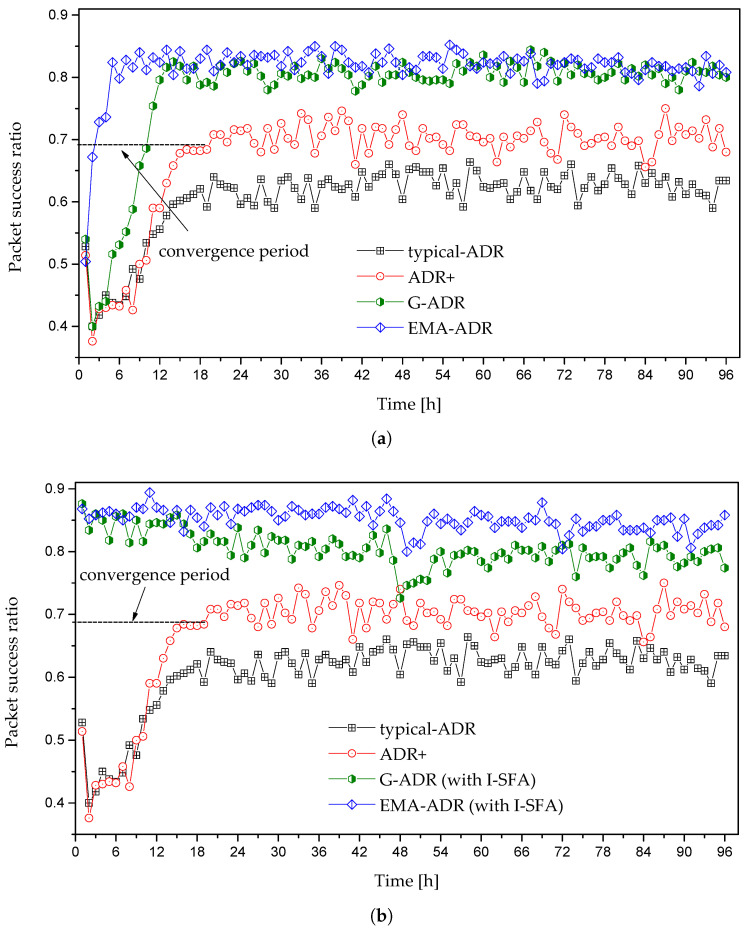
Convergence period and PSR of static EDs with N = 500: (**a**) initial allocation of SF with 12, and (**b**) initial allocation of SF with 12 (for ADR and ADR+) and initial SF allocation with I-SFA (for G-ADR and EMA-ADR).

The convergence period and PSR of static EDs during a simulation period of 4 days is shown in Figure 8. All of the schemes begin transmission with SF = 12 and TP = 14 dBm, as indicated in Figure 8a. Figure 8a,b show the amount of time for the EDs to reach a steady SF and PSR (i.e., convergence period), whereas both typical ADR and ADR+ require 20 h in converging to a stable state. The primary reason for this high convergence period is that the frequency of a typical ADR (NS-managed ADR) is entirely arbitrary, which is activated after *M* UL packets. The changes in the ED-managed ADR occur in time in steps of ADR_ACK_DELAY × uplink_period [18]. This is a time-consuming process and, thus, yields a high convergence period. In general, an ED-managed ADR is intended to maximize DL traffic flexibility, restricted by the duty cycle limitations that are imposed by LoRaWAN. This helps the EDs to reestablish reliable communication links by steadily increasing the SF (SF<12). This flexibility increases the convergence period in the worst case. For example, an ED is presently employing a lower SF than needed to deliver a packet to the nearest GW successfully.

By contrast, G-ADR follows a similar trend with a convergence period of 14 h along with a better PSR when compared to both a typical ADR and ADR+. Moreover, EMA-ADR outperforms the other schemes in terms of both the convergence period (i.e., 3 h) and PSR. As the reason for the quick convergence, the EMA filter resists against the rapid changes in the SNR of *M* packets and acts as a local averaging function. Thus, the proposed EMA-ADR scheme attains a higher PSR that is related to typical ADR and ADR+, as shown in Figure 8a. Figure 8b presents another scenario in which G-ADR and EMA-ADR are employed while using the I-SFA scheme. There is no convergence period when I-SFA is jointly utilized with both G-ADR and EMA-ADR. This is because I-SFA assigns a suitable SF to the EDs based on $P_{r}$. Table 4 highlights the detailed convergence periods of different EDs in hours for static EDs.

#### 4.3.2. Mobile EDs Scenario

**Figure 9:**
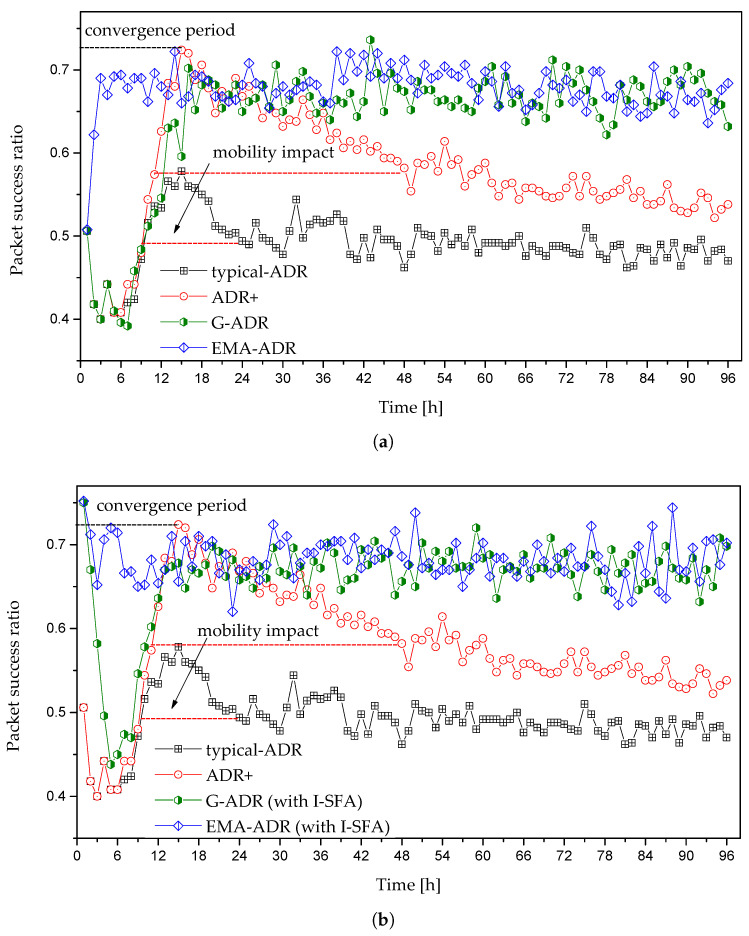
Convergence period and PSR of mobile EDs with N = 500: (**a**) initial allocation of SF = 12, and (**b**) initial allocation of SF = 12 (for ADR and ADR+) and initial SF allocation with I-SFA (for G-ADR and EMA-ADR).

Figure 9 shows the convergence period and PSR of mobile EDs, where both typical ADR and ADR+ require 15 h and 14 h, respectively. Further, in Figure 9a, we can observe two types of convergence period (in the case of typical ADR and ADR+), which are the initial convergence period and convergence period caused by the mobility. The initial convergence period occurs owing to a high SF (because all EDs initially start transmitting packets with SF = 12). However, after a short period of stability, PSR decreases, and both typical ADR and ADR+ suffer from a convergence period. This convergence period is caused when a mobile ED receives a LinkADRreq MAC command containing SF and/or TP from the NS; the propagation environment might have been drastically altered, resulting in massive packet loss [16,32].

In Figure 9a, G-ADR and EMA-ADR take 16 h and 3 h for the initial convergence period, respectively, but do not suffer a second convergence period. This is because both of the proposed schemes employ filters, which helps to reduce the convergence period and improve the PSR. As shown in Figure 9b, both G-ADR and EMA-ADR use I-SFA as an initial SF assignment during deployment. Unlike a static scenario, both the proposed G-ADR and EMA-ADR with the I-SFA scheme have a convergence period of 13 h and 3 h. In the case of G-ADR (with I-SFA), mobility has a considerable impact on the convergence period because the newly adopted parameters (i.e., both SF and TP) do not provide assurance and efficient communication. In such a case, the propagation environment might have changed radically by the time LinkADRreq command reaches the mobile ED. Hence, a new packet from this ED with recently adopted parameters can be lost. However, EMA-ADR (with and without I-SFA) performs exceptionally well under the mobility conditions, resulting in a reduced convergence period and high PSR. The detailed convergence periods of different mobile EDs in hours are shown in Table 5.

### 4.4. Average PSR Analysis

In this study, the PSR is defined as when both the confirmed uplink packet and the equivalent downlink packet (i.e., ACK) are correctly received in one of the available transmission attempts [36].

#### 4.4.1. Static EDs Scenario

**Figure 10:**
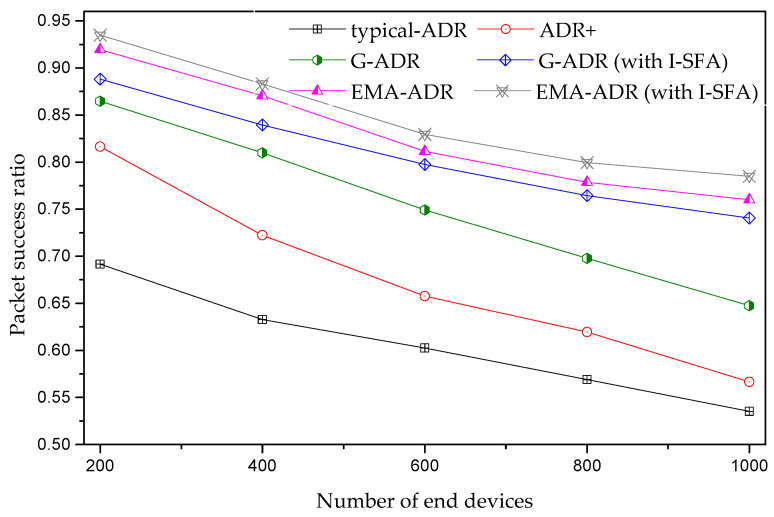
Average PSR of static EDs (uplink period = 24 packets/day, total simulation time = 4 days).

The average PSR for a different number of static EDs is presented in Figure 10. Here, Figure 10 shows a decreasing trend in PSR with an increasing number of EDs in a confirmed mode due to high interference among the SFs when packets are transmitted with a high SF. These higher SFs (e.g., 11 and 12) are weak to interference, owing to the high ToA, which negatively influences the capacity of the communication channel [37,38,39,40,41]. Thus, retransmissions from the EDs are increased, which results in substantial congestion and massive packet loss. However, the proposed EMA-ADR with and without the I-SFA scheme outperforms the other schemes in terms of PSR because EMA-ADR frequently changes the SF and TP parameters by employing a low-pass filter and averaging function. In addition, for a similar scenario, as presented in Figure 10, Table 6 shows the average PSR improvement for ADR+ and the proposed schemes when compared to a typical ADR.

#### 4.4.2. Mobile EDs Scenario

**Figure 11:**
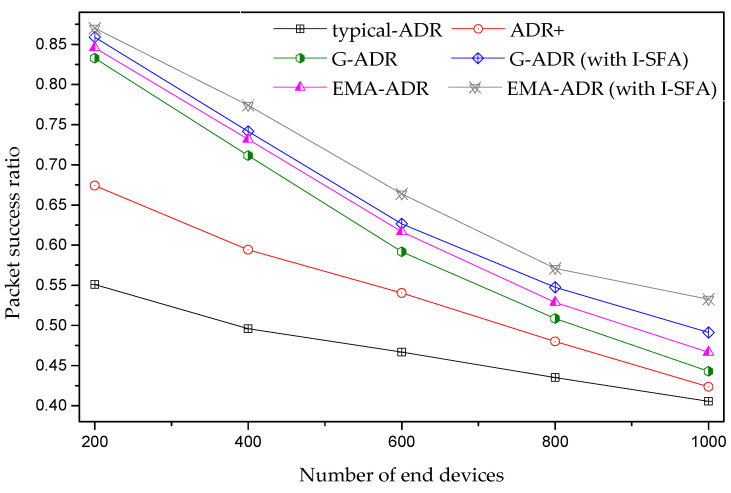
Average PSR of mobile EDs (uplink period = 24 packets/day, total simulation time = 4 days).

Figure 11 shows an analysis of the average PSR for a different number of mobile EDs. The mobility of the EDs has a high impact on the PSR because the mobility causes frequent changes in the topology, influencing the signal strength between an ED and a GW. As a result, the link budget used at the previous location after the ED movement would no longer be valid. Thus, these EDs are required to alter their SF due to the received signal strength variations. However, when the NS changes the SF and TP (in a typical ADR and ADR+), these parameters are no longer valid owing to ED mobility. As a result, a packet transmitted from these EDs is lost because of arriving under the sensitivity at the GW.

Another reason for this massive packet loss in Figure 11 is due to the saturated receiver. Under this situation, a packet transmitted by the ED reaches the GW with adequate power; however, all of the available parallel reception paths are already busy in the reception of other incoming packets, resulting in packet loss. A GW can decode as many overlapping packets as the number of paths listening to that channel. In other words, when a packet arrives on a given channel, it will “lock” only one receive path. By contrast, other reception paths remain available to receive other incoming packets. Therefore, when a packet arrives at a channel, and there are no available receive paths, the packet is lost. Moreover, we adopt an assumption from [35] that if the GW is in a receive state and is asked by the NS to forward a DL packet to an ED, it will give up the incoming packet reception and transmit the DL packet. Both G-ADR and EMA-ADR (with and without I-SFA) manage a high PSR when compared to a typical ADR and ADR+. Furthermore, for a scenario similar to that in Figure 11, Table 7 shows the average PSR improvement for ADR+ and the proposed schemes when compared to typical ADR.

### 4.5. Average Energy Consumption Analysis

In this study, we compute the energy consumption as the total energy spent by EDs during the simulation time divided by successfully received packets. This study assumes a state-based (i.e., transmit, receive, standby, and sleep) energy consumer module [23]. The energy consumption in LoRaWAN mainly depends on the amount of time that is spent by a LoRa radio in a particular state. In this study, we have utilized the energy consumer module, as shown in [42] and the Semtech SX1272/73 datasheet with a supply voltage of 3.3 V [26].

#### 4.5.1. Static EDs Scenario

**Figure 12:**
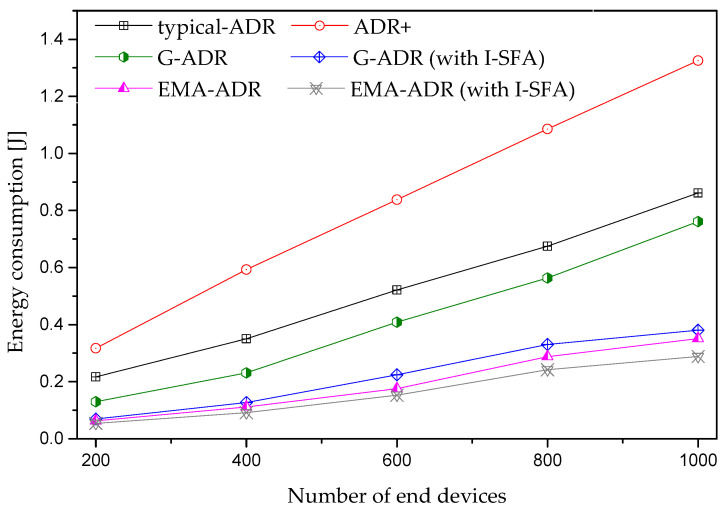
Average energy consumption for static EDs (uplink period = 24 packets/day, total simulation time = 4 days).

In general, the energy consumption of all schemes in the confirmed mode shows an increasing trend as the number of EDs increases, as shown in Figure 12. However, in the proposed schemes, the energy consumption is lower than the typical ADR and ADR+, owing to the small number of retransmissions. However, in typical ADR and ADR+, many EDs transmit packets with high parameters, including SF = 12 and TP = 14 dBm. The maximum number of packets is lost due to increased interference, resulting in EDs retransmitting packets with higher settings. Therefore, high energy consumption is observed for typical ADR and ADR+, because the transmit energy consumption is primarily based on the values of SF, TP, and ToA, and the retransmissions [23].

#### 4.5.2. Mobile EDs Scenario

**Figure 13:**
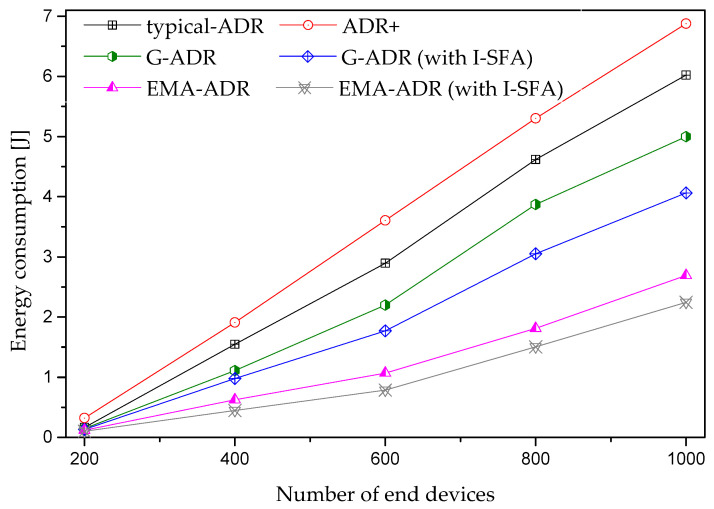
Average energy consumption for mobile EDs (uplink period = 24 packets/day, total simulation time = 4 days).

Figure 13 shows the average energy consumption of the proposed schemes compared with typical ADR and ADR+. Overall, the energy consumptions of the proposed G-ADR and EMA-ADR (with and without I-SFA) are lower because of the higher PSR. Generally, the energy consumption of all schemes shows an increasing trend as the number of EDs increases owing to multiple retransmission with high SF and TP [32]. When packets are transmitted with higher SFs, it causes a high interference due to the high ToA. Because higher SFs are highly susceptible to interference, they can negatively affect the energy consumption [37,38,39,40,41].

## 5. The Adaptation of Proposed Schemes in a LoRaWAN Deployment

The typical ADR of LoRaWAN requires the operation of both the NS and the ED. In general, to deploy the new ADR in the ED, the so-called firmware update process must be performed. These firmware updates are usually performed through a wired connection, such as serial communication, to the target ED. However, because of the nature of the massive LoRaWAN, this method is challenging. Therefore, STM, one of the LoRa Mote makers, supports firmware update over the air (FUOTA) [43,44]. FUOTA is an excellent approach to deploy the ADR algorithm to the ED. However, our proposed methods (i.e., G-ADR and EMA-ADR) are the improved ADRs on the network server-side and do not require ED update technology like FUOTA. Therefore, the proposed ADRs can be deployed by updating the server itself as part of its maintenance work.

## 6. Conclusions

LoRaWAN allocates resources to the EDs (such as SF and TP) through NS-managed and ED-managed adaptive data rate (ADR) methods. However, the ADR is severely affected by the lack of agility to adapt to the variable channel conditions. Thus, it has a high convergence period and requires several hours to days to converge to a stable and energy-efficient state of communication. During the convergence period, the typical ADR suffers from a massive packet loss caused by interference and packets arriving under the sensitivity at the GW. Therefore, to reduce the convergence period and improve the PSR (for both static and mobile IoT EDs), we proposed two NS-managed ADRs (G-ADR and EMA-ADR). Both of the proposed schemes operate as a low-pass filter to resist the rapid changes in the SNR of *M* packets. Through NS-3 simulation experiments, we extensively analyzed the proposed schemes as compared to a typical ADR and ADR+. We showed that a typical ADR and ADR+ both suffer from a high convergence period owing to the time-consuming process and poor adaptation of the SF. In contrast to the typical ADR method, the proposed methods reduce the convergence period, energy consumption, and enhanced the PSR for both static and mobile EDs. We further remark that the proposed methods are highly suitable for IoT-based static and mobile applications requiring a low convergence period, high PSR, and reliability without sacrificing a high energy consumption.

## Figures and Tables

**Table 1:** Sensitivity and required signal-to-noise ratio (SNR) of EDs and GW with 125-kHz mode [25,26].

SF	GW Sensitivity ($S_{g}$) [dBm]	ED Sensitivity ($S_{e}$) [dBm]	SNR [dB]
12	−142.5	−137.0	−20
11	−140.0	−135.0	−17.5
10	−137.5	−133.0	−15
9	−135.0	−130.0	−12.5
8	−132.5	−127.0	−10
7	−130.0	−124.0	−7.5

**Table 2:** Simulation parameters.

Parameter	Value
Simulation time [days]	4
GW	1
λ	24 packets/day
Packet length [bytes]	51 [24]
UL packet transmission limit	8
Path loss exponent	3.76 [33]
Propagation model	log-distance
Shadowing	de-correlation distance = 110 m [34] and
	variance = 6 dB [35]
ED movement speed [m/s]	0.5∼1.5
Transmit power [dBm]	2∼14
Frequency region	EU-868
Channel bandwidth [kHz]	125
Coding rate	4/8

**Table 3:** Final SF assignment percentage of static EDs (with starting SF = 12, N = 500, uplink period = 24 packets/day, and total simulation time = 4 days).

Scheme	ADR	ADR+	G-ADR	G-ADR (I-SFA)	EMA-ADR	EMA-ADR (I-SFA)
SF 7	25.8%	14%	25.4%	24%	25.8%	25.2%
SF 8	8.4%	6.2%	6.4%	10%	10.8%	6.6%
SF 9	11%	7%	12.2%	13.6%	13.6%	12%
SF 10	16%	9.8%	14.4%	21.8%	24.2%	14%
SF 11	24%	11.8%	23.4%	19.8%	19.4%	23.4%
SF 12	14.8%	51.25%	18.2%	10.8%	6.2%	18.8%

**Table 4:** Convergence period in hours for static EDs (with starting SF = 12, uplink period = 24 packets/day, and total simulation time = 4 days).

N	ADR	ADR+	G-ADR	G-ADR (I-SFA)	EMA-ADR	EMA-ADR (I-SFA)
200	20	18	15	0	3	0
400	21	19	15	0	3	0
500	20	20	14	0	5	0
600	18	20	16	0	6	0
800	28	28	26	0	11	0
1000	40	39	37	0	19	0

**Table 5:** Convergence period in hours for mobile EDs (with starting SF = 12, uplink period = 24 packets/day, total simulation time = 4 days).

N	ADR	ADR+	G-ADR	G-ADR (I-SFA)	EMA-ADR	EMA-ADR (I-SFA)
200	22	21	15	16	3	0
400	23	20	17	15	6	0
500	15	14	16	13	3	3
600	13	14	14	13	3	3
800	20	21	22	15	4	3
1000	29	23	17	17	4	3

**Table 6:** PSR improvement for static EDs.

N	ADR	ADR+	G-ADR	G-ADR (I-SFA)	EMA-ADR	EMA-ADR (I-SFA)
200	-	+12.5%	+17.3%	+19.7%	+22.8%	+24.3%
400	-	+8.9%	+17.7%	+20.7%	+23.8%	+25.0%
600	-	+5.5%	+14.7%	+19.5%	+20.9%	+22.7%
800	-	+5.1%	+12.9%	+21.5%	+21.0%	+23.0%
1000	-	+3.1%	+11.2%	+20.5%	+22.5%	+25.0%

**Table 7:** PSR improvement for mobile EDs.

N	ADR	ADR+	G-ADR	G-ADR (I-SFA)	EMA-ADR	EMA-ADR (I-SFA)
200	-	12.3	28.2	30.8	29.5	31.9
400	-	9.8	21.6	24.6	23.6	27.8
600	-	7.4	12.5	16.0	15.0	19.7
800	-	4.5	7.3	11.2	9.4	13.6
1000	-	1.8	3.8	8.6	6.1	12.7

## Abbreviations

The following abbreviations are used in this manuscript:

ACK	Acknowledment
ADR	Adaptive Data Rate
DL	Downlink
ED	End Device
EMA-ADR	Exponential Moving Average ADR
G-ADR	Gaussian Distribution based ADR
GW	Gateway
I-SFA	Initial Spreading Factor Assignment
IoT	Internet of Things
LoRaWAN	Long-Range Wide Area Network
LPWAN	Low-Power Wide Area Network
MAC	Media Access Control
NS	Network Server
PSR	Packet Success Ratio
RX	Receive Window
SF	Spreading Factor
SNR	Signal-to-Noise Ratio
ToA	Time-on-Air
TP	Transmit Power
UL	Uplink
